# Comprehensive motivational framework to drive paddy farmers towards pluriactivity

**DOI:** 10.1038/s41598-023-35368-1

**Published:** 2023-05-20

**Authors:** Hadi Moumenihelali, Enayat Abbasi, Mostafa Karbasioun

**Affiliations:** 1 Department of Agricultural Extension and Education, Faculty of Agriculture, Tarbiat Modares University, Tehran, Iran; 2 Department of Agricultural Development, Faculty of Agriculture, University of Shahrekord, Shahrekord, Iran

**Keywords:** Psychology and behaviour, Socioeconomic scenarios, Sustainability

## Abstract

Pluriactivity is a livelihood strategy in line with rural resilience. It is a phenomenon of farming in conjunction with other gainful activities. In pluriactivity, the desire and motivation of setting up an extra business and taking necessary actions are crucial. Therefore, the main aim of this study was to identify the underlying components of pluriactive paddy farmers’ motivation and also the factors affecting them. The study was implemented based on the quantitative data obtained from 182 pluriactive paddy farmers. The results of the exploratory factor analysis accentuated that there are three components in each of the pull and push typologies. The components related to pull motivation included personal aspirations and pursuit (C1), proper conditions and facilities (C2) and growth and service markets (C3). Likewise, the components related to push motivation included financial status and job creation improvement (C4), uncertainty and risk mitigation (C5) and paddy farming economic enhancement (C6). Also, it was revealed that two motivational components of personal aspirations and pursuits (C1) and financial status and job creation improvement (C4) are attributed to paddy farmer’s age of the pluriactivity initiation and also the farm size variables. It is imperative to consider both pull and push strategies in directing paddy farmers towards extension and development of pluriactivity in rural areas to enable farmers achieve sustainable livelihood in line with rural resilience.

## Introduction

Entrepreneurship is regarded as the catalyst to achieve economic and social development objectives, including growth, innovation, employment, and equity^1^. So that it can be assumed as an important source of income and employment for societies^2^. Paying attention to the livelihood of paddy farmers) and achieving a standard life in Watershed of the Haraz Plain (WHP), as about 60% of the total population of farmers in this region, is vital in supporting rural resilience in northern Iran. Neglecting this issue has led to significant migration of paddy farmers to urban areas, which in turn, has resulted in rural decline. In a 5-year period (2011–2016), approximately 13,000 of the villagers have migrated to the cities and in the same period, roughly 9% of the lands under the rice cultivation in this area has been reduced^3^. Rural regions have the potential to perform a variety of functions. Hence, it is important to use the capacity of rural regions to deal with changing external status in a way that a standard living is retained^4^. Pluriactivity, as a manifestation of rural entrepreneurship and in line with rural resilience, is the *heart of livelihood strategies* in rural areas^5^; while, the motivation is considered as a driving force of pluriactivity phenomenon^6^.

Agricultural households that focus solely on producing one crop are vulnerable to environmental stresses and shocks^7^. It should be noted that such vulnerability, results from households’ social, economic, political, cultural, and environmental conditions. The concept of vulnerability has two components: Sensitivity; and adaptive capacity. Sensitivity describes a household’s susceptibility to a undesirable environment event, depending on their livelihood conditions. Adaptive capacity is the ability of a household to adjust with, reduce, or mitigate the impacts of undesirable environmental events^8^. Therefore, vulnerability to environmental stresses and shocks is intrinsically related to a household’s ‘livelihood’, defined as the assets (both material and social resources), capabilities, and activities required to sustain a household^9^. For example, a household whose livelihood is poorly diversified will be more vulnerable to environmental shocks; because, it will lack livelihood alternatives that might withstand extreme environmental events^10^. In particular, rural livelihood diversification is essential for reducing vulnerability to undesirable environmental events^11,12^. Rural livelihood diversification comprises of upholding and embracing a varied portfolio of activities to survive and cure living norms^13,14^. This portfolio encompasses on-farm and off-farm activities -called pluriactivity- that assist increase revenue, improve propertys, and make resilience to periods of off-peak agriculture production and risks, including diversification and management practices^13–16^.

Rural resilience plays an important impress in maintaining rural stability and people’s living norms in the face of threats and environmental stresses and shocks^17^. In social and economic frameworks, resilience is understood as the ability to admit change, with a susceptibility to adapt seamlessly to largely exogenous phenomenons in a form of sustainable adaptation^18^. Giving the reality that alteration is ever occurring, resilience is specified as the ability of a system to absorb disturbance and still sustain its basic operation and structure^19^. The multifunctionality of rural regions and their likely resilience is the focus of Wilson^20^, who finds that places with strongly developed economic, social and environmental capital are likely to be more resilient than places where only one, or none, of these factors is present. Wilson^20^ integrates the components by noting that rural communities to be resilient in economic, social and environmental terms, they need to develop strong multifunctional characteristics. Several studies have been conducted by various researchers in different fields as well as economics, geography and sociology focused on pluriactivity or diversification. Most scholars have explained the term of pluriactivity/diversification, their varieties and also their effects on different geographic locations of the world^21–30^.

In the context of the entrepreneurship in general and setting up and developing rural business in particular, this statement suggests that the success largely depends on people’s willingness to become entrepreneurs^31^. To formulate a successful policy, the policy-makers are expected to consider the behavior of the target population^27^. Therefore, a successful agricultural program should be built on a profound understanding of farmers' behavior^32^. Several authors have implied the need to clearly understand the motivation of entrepreneurs in order to comprehend their behavior^31,33–35^. Some of scholars have referred to villagers’ motives in pluriactivity/diversification^22,23,25–27,29^. In the studies of pluriactivity/diversification, the individuals' motivations are very different in starting a business and therefore their nature and role cannot be considered the same. They need to be categorized based on these two factors. In this regard, using motivational theories can be very useful. In the literature of pluriactivity/diversification, pull and push motives have not been assessed separately so far. It is why individual motivation is rarely clear-cut in pluriactivity/diversification.

Due to the characteristics of rice production and its seasonality nature and availability of farmers and their family members at certain times of the year (in the second half of the year) in the watershed of the Haraz plain of Iran, pluriactivity could be presumed as one of the most appropriate rural development strategies in line with rural resilience. It is strongly recommended for using the capacities of the region, increasing farmers' income, and providing supplementary employment for them. Therefore, recognizing their motivational characteristics as the drives of pluriactivity development, can affect various aspects of their life towards sustainable livelihood and rural resilience. in sum, basic questions of the research are as following:

- Based on the elements of pull and push, what are the paddy farmers' motives to be pluriactive?
- What effects the paddy fields and paddy farmers’ features have on pull and push motives?

From a theoretical point of view, our work contributes to comprehensive understanding of pull and push motivational theory in the pluriactivity of the paddy farmers. From a managerial perspective, our work provides practical insights for policy and decision-makers in provision of the facilities required for the paddy farmers to move better towards pluriactivity.

In throughout the article, first the theoretical background is presented in the fields of rural resilience, pluriactivity and farmers’ motives and then the data collection methods, results, discussion, conclusions, and policy implications are expressed. In the results' section, the importance of pull and push motives is evaluated. In the second step, using exploratory factor analysis, the underlying components of each of the pull and push typologies were extracted. In the third step, the effects of paddy farmers' and paddy fields' features on pluriactivity motivation are measured. The fourth step affords a comprehensive framework as a driver for rural pluriactivity promotion.

## Theoretical background

### Rural resilience

The term of resilience has been emerged as a new concept to raise the comprehension of interrelationships between ecological and social dimensions in the late last century^36–39^. Rural resilience represents rural areas’ ability to tackle with its intrinsic economic, ecological, and cultural vulnerability. Based on this view, ecological, economic, and cultural systems are increasingly intertwined; hence, the interaction of these systems is increased in terms of intensity and scale^4^.

While the decline of some rural societies has become an obvious process in the world, the promotion of rural resilience plays a major role in sustainable rural development^40^. Rural areas as the center of agricultural production in developing countries, including Iran, play an essential role in ensuring food security and as a result, independence of related countries^41,42^. It should be noted that unbalanced policies in favor of urban areas in some countries could cause a difference in the standard of living in urban and rural areas, and this issue has sloped urban–rural relations and intensified rural decline in the long term^40^. Rural areas have plenty of economic potentials in which recognizing their entrepreneurial opportunities and subsequently a proper program planning for these regions could generate a dynamic and diverse economy in mentioned territories^41^. One of the most significant strategies for applying these potentials is Pluriactivity^43^. This approach could largely prevents rural decline and subsequently accelerate revitalization of rural areas.

### Pluriactivity

It is widely accepted that the risk is an integral part of agricultural production; hence, most farmers consider diverse strategies to reduce negative intervening factors^44^. Ilbery^45^ and Abera et al.^46^ argued that the business diversity is an undeniable necessity for farmers to be able to overcome the risk. One of these management strategies is pluriactivity. The pluriactivity is known as an attractive phenomenon in farmers’ activities in general, and according to economic history, farmers have always tried to get benefit from other income sources along with their farming tasks^29^. It is mostly defined as an off-farm economic activity engaged by the farmer and other household members, typically for the risk-reduction and farm income’s supplementation purposes^47^. In pluriactivity, the goal is not to focus only on a particular job, but rather on the overall household income^29^. In other words, the accomplishment of the farming along with other profitable activities (whether on-farm or off-farm), is defined as pluriactivity. According to Blad^29^, the pluriactivity is used to denote situations in which the individuals or households combine farm and non-farm employment or revenue streams, regardless of their origins or locations. Consequently, the concept of pluriactivity refers to the household farming as an economic unit, in which all household members contribute to its income through engagement in agricultural and/or non-agricultural activities, whether on-farm or off-farm^22^. Sometimes, moving toward pluriactivity might be due to the lifestyle choices or broader non-economic goals^47–49^. Previous studies have demonstrated that the income diversification is rising from on-farm/non-farm or on-agriculture/non-agriculture (pluriactivity) actions used by farmers to survive and even prosper in today’s changing agricultural climate as a sustainable strategy^21,24,28,50,51^.

At the district program level, the pursuit of entrepreneurship is vital for the development of economically strong regions^52^. The literature discloses a changing variety of diversification as the new challenges and opportunities over the time. Vik and McElwee^53^ and De Rosa et al.^54^, categorized diverse farmers’ activities into four classes: (i) on-farm and farm-related (e.g. fire wood); (ii) tourism (e.g. lodging or accommodation, adventures, tours, guiding, etc.) and social farming (e.g. green care, relieving, etc.); (iii) off-farm and farm-related (e.g. machinery contracting, haymaking, snow clearing, etc.); (iv) off-farm and farm-diverse (e.g. consulting and accounting services). Morris et al.^55^, classified different activities based on four general strategies including resource maximizing (e.g. seeking a range of income streams), farm-focused (e.g. business focusing largely on the farm), lifestyle farmers (e.g. developing off-farm income streams), and passive farmers (e.g. displaying limited engagement with grant-focused opportunities). Iranian Interior Ministry^56^, has considered different business types in rural regions into three sections: (a) agriculture (e.g. livestock and poultry); (b) industry (e.g. garment production); (c) services (information and communication technology services).

### Paddy farmers’ motives

The majority of motivation theories concentrate on the determinants and processes underlying the optimal behaviors, volitional activities, and development of intentions^33^. The theories of entrepreneurship motivation can be generally divided into two groups: driving and incentive theories^57^. The driving theories underline the fact that there is an internal need (e.g., achievement or autonomy) with the power of motivating individuals to start a new venture in order to reduce the subsequent tension. On the other hand, the incentive theories argue that the people are motivated to do the things because of external rewards. For example, entrepreneurs could be motivated by a mixture of incentives such as the suppleness, revenue, or prestige^58^. In addition, general consensus is that entrepreneurs are either pushed into entrepreneurial activities by dissatisfaction with their previous job situations or pulled into them by the job and life satisfaction^27^. According to Hansson et al.^27^, the push/pull literature would suggest that diversification (pluriactivity) is “opportunity-driven” (pull factor) or “necessity-driven” (push factor). The term pull can be used in a situation that new activities are started because a farmer who has perceived a business opportunity, wants to implement a good business idea, or to reallocate existing resources and/or gain business growth. The term push can be used for a situation in which a farmer has to diversify his/her income sources in order to become self-employed, to provide secure family income, or to mitigate the risks arising from changes in the market situation. The interest of the push/pull framework lies in understanding the motives to move from one stage to another, rather than general goals and values that may underlie the decision-making process more continuously. Therefore, the push/pull literature should be distinguished from other literature describing farmers’ goals and values at a more general level^59,60^. According to Hansson et al.^27^, there has been a common agreement as to the farmers’ motivation analysis in rural entrepreneurship (pluriactivity and diversification) based on the two dimensions of push and pull factors.

As already mentioned, many studies have focused on the concept of “the pluriactivity/diversification by farmers”. Nevertheless, only few studies paid attention to farmers' motivation in starting a business. The researchers have mainly referred to the most important motives of farmers to start a business, which is pluriactivity or diversification.

Barbieri and Mahoney^26^, used an exploratory factor analysis to classify 19 reasons for setting up a business by farmers in six components including uncertainty and risk mitigation (URM), growth and service markets (GSM), enhanced financial condition, individual aspirations and pursuits, revenue enhancement, and family ties. They investigated the relationship among various entrepreneurs and farm characteristics and the goal pursuit components. Hansson et al.^27^, employed 15 reasons for setting up a business to categorize in two components. These components incorporated to “business development to reduce the risk and to use idle resources” and “business development for social and lifestyle reasons”. Finally, they showed that the family situations were effective on farmers’ motivation in starting new ventures. Bowler et al.^61^, identified some factors such as: sustaining or increasing the income generated by the farm business, reactions toward a market opportunity, better exploitation of an under-utilized farm resource, and employment generation for a family member as the main reasons for the development of alternative enterprises by English farmers. Sofer^22^, uncovered that the decrease in agricultural income, exploitation of non-agricultural vocation, unused premises or lands, unwillingness to do agriculture practices, accumulated agricultural debts, availability of wage labour, excess labour resources within the household, lack of younger generation and pressures of external entrepreneurs, were presumed as the main reasons for the farmer's household to become pluriactive. Nickerson et al.^62^, tested 11 motives for diversifying activities by farmers. The factor analysis resulted in three factors: social, economic, and external influences. Also, the results of the cluster analysis showed that 61% of the respondents diversified their activities for economic reasons; while, 23% diversified for external operation reasons, and 16% diversified for social, economic, and external reasons. Taylor and McClintock^23^, identified the criteria of need for extra or regular income, insufficient income to sustain living standard, persuasion by others to take up a job, following a particular profession or occupation, choosing a particular lifestyle, and making social off-farm contact with other people as the main reasons of taking multiple jobs by the New Zealand men and women farmers. McGehee and Kim^63^, used an exploratory factor analysis to classify 11 motives for agri-tourism business in three components based on Weber's theory. These components included formal motivations, formal-substantive mix motivations, and substantive-formal mix motivations. Blad^29^, presented that insufficient farming incomes, the desire to achieve a higher living standard, closeness of the workplace, the desire to try a new situation, the desire to utilize qualifications, the desire to fulfill dreams or passions, and the availability of free times were considered as the most important rationales for the pluriactivity of farming families.

**Figure 1:**
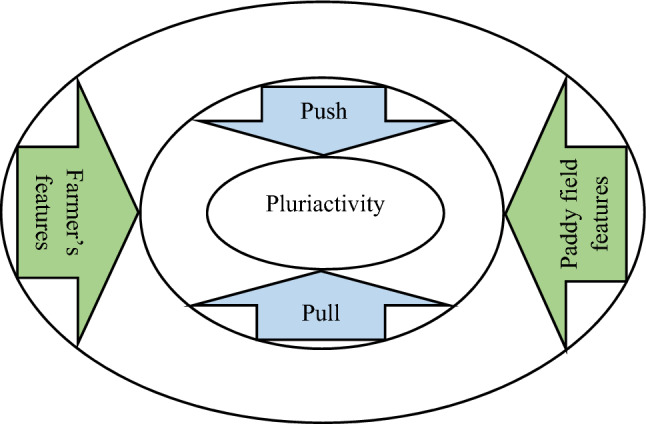
Theoretical framework.

According to the literature and to the best of our knowledge, despite examining farmers' motivation for pluriactivity/diversification in the previous studies, no inclusive empirical analysis of the pull and push theory in relation with farmers’ response to starting an extra business, has been accomplished yet and this study aimed at resolving this gap. Therefore, unlike other research in this area, current study evaluated the typologies of pull and push separately (Fig. 1). To investigate the difference between two elements of pull and push in motivating farmers to start an extra business, the purposes of this study are as below:

1. Examining relative importance of the pull and push motives.
2. Extraction of underlying components of the pull and push motives.
3. Determining the effect of paddy fields and paddy farmers’ features on pull and push motives.
4. Designing a comprehensive motivational framework based on the factors affecting launching an additional enterprise.

## Materials and methods

### Geographical context

**Figure 2:**
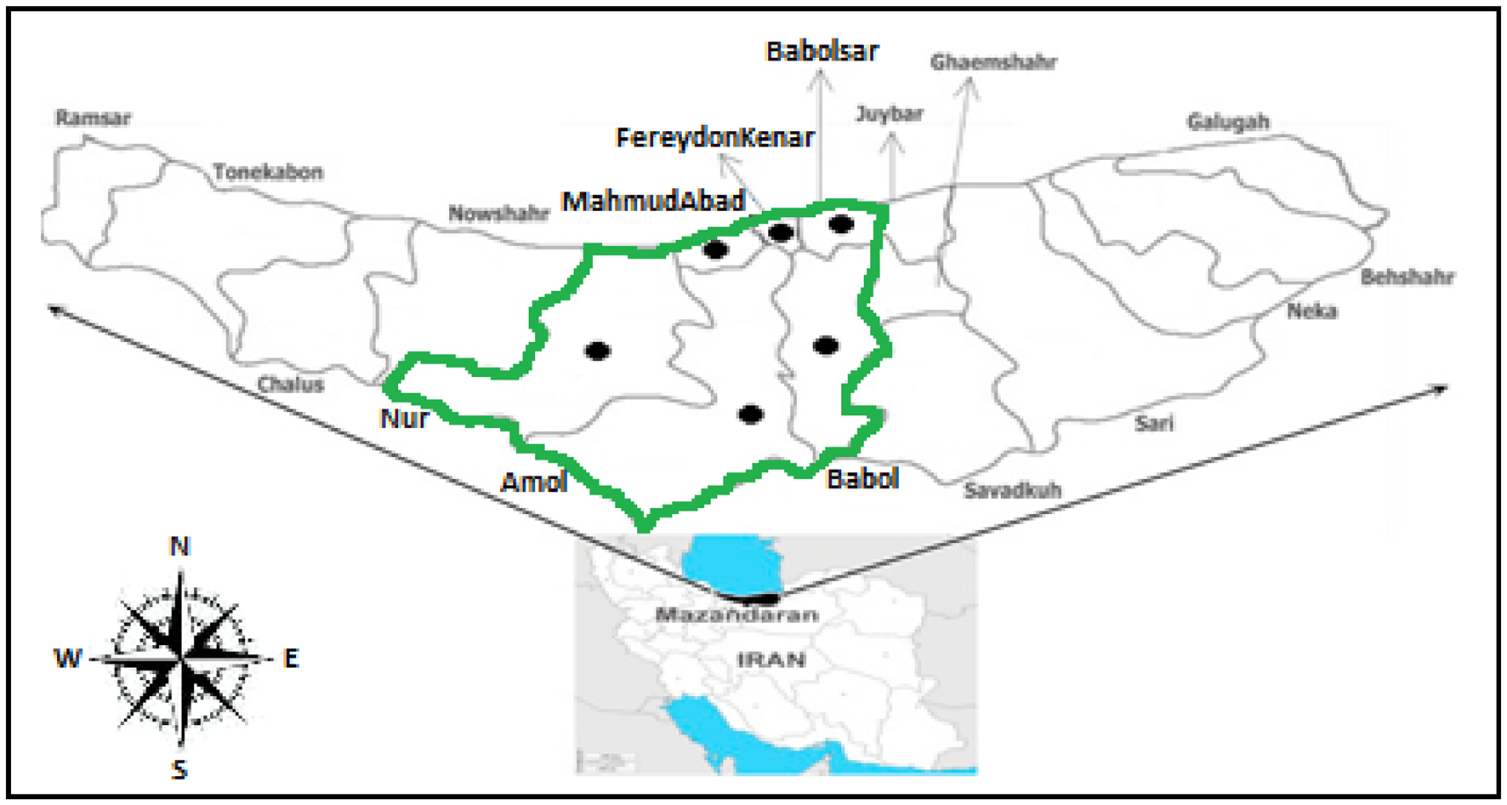
A schematically view of Iran, Mazandaran Province and WHP (Black filled circle). (Created using miniPaint Application. URL link: https://viliusle.github.io/miniPaint/).

The WHP is located at the central part of the Mazandaran province, Iran. This region includes Amol, Babol, Babolsar, FereydonKenar, MahmudAbad, and Nur (Fig. 2). Mazandaran province has the highest paddy cultivating area (37%) and rice production (38.4%) in Iran. In this regard, the WHP is one of the high-quality plains for paddy cultivation in Iran. This plain has the highest rice farming area (58.65%) in Mazandaran province^64^.

## Research method

The present research was a quantitative study in terms of nature and benefited from a survey methodology. In addition, it was a non-experimental type in terms of the control degree over the variables, and an applied study in terms of its objective. Hence, the results can be used by program planners and authorities involved in agriculture and rural development.

### Sampling and surveying

The study population was all paddy farmers in the WHP, who had set up at least one rural business along with their paddy farming (paddy farming as the main career) and were the manager of their businesses (= pluriactive paddy farmers (PPFs)). In many research situations including the present one, it is very difficult to count demographics as a basic requirement used for the probability sampling method because there is no proper database from the number of statistical population. In these cases, the researchers use non-probability sampling method^65,66^. The snowball sampling technique is a non-probability sampling method that allows identification of the best samples for a certain study^26,67^. Using this technique, a number of the PPFs were identified with the help of rural experts. At the next step, with the help of these PPFs, other PPFs were recognized. Finally, 198 PPFs were recognized. Using the census method, all of them were taken into consideration as the statistical sample of this research, and 182 participated in the study (Table 1).

**Table 1:** Distribution of questionnaires in six regions of the WHP.

Regions	Completed questionnaires (n)
Amol	30
Babol	32
Babolsar	30
FereydonKenar	30
MahmudAbad	30
Nur	30
Total	182

Thus, 182 PPFs reported that they were in charge of at least one business along with their paddy farming system at least over the last 2 years and were therefore included in the study sample. Out of this number, 128 (68.7%) had a business in agricultural sector (in addition to rice cultivation), 11 (6%) had a business in the rural industrial sector, and 46 (25.3%) had an enterprise in the rural service sector. The PPFs’ age levels were categorized as below: less than 35 years, 36–55 years, and more than 55 years. Most participants (70.88%) were middle-aged and male (95.5%). The mean of paddy farming experiences was 20.01 years. The mean age of pluriactivity initiation was 31.83 years. The mean of paddy field size was 1.20 ha. The educational level of 39% of the studied PPFs was high school. The majority (51.6%) of respondents had less than 2 years of experience at the beginning of pluriactivity as the owner. The PPFs’ educational level at as the owner at the beginning of pluriactivity was appropriate.

## Research tool and statistical methods

The main study instrument was a researcher-made questionnaire. The questionnaire consisted of two sections. The first section focused on demographic and professional characteristics of the PPFs including the age that the pluriactivity was initiated, paddy farming experience, paddy field size, experience at the beginning of pluriactivity as the owner and educational level. The variables representing demographic and professional characteristics of the PPFs were extracted based on the literature^28,30,33,55^ and research site characteristics. The second section was allocated to the motives of the PPFs. The research included twenty motives in two typologies of pull (13 items) and push (seven items) representing a wide spectrum of economic, social, intrinsic and market-related motives reported by the literature as the factors stimulating Pluriactivity^22,26,27,29,62,63^.

**Figure 3:**
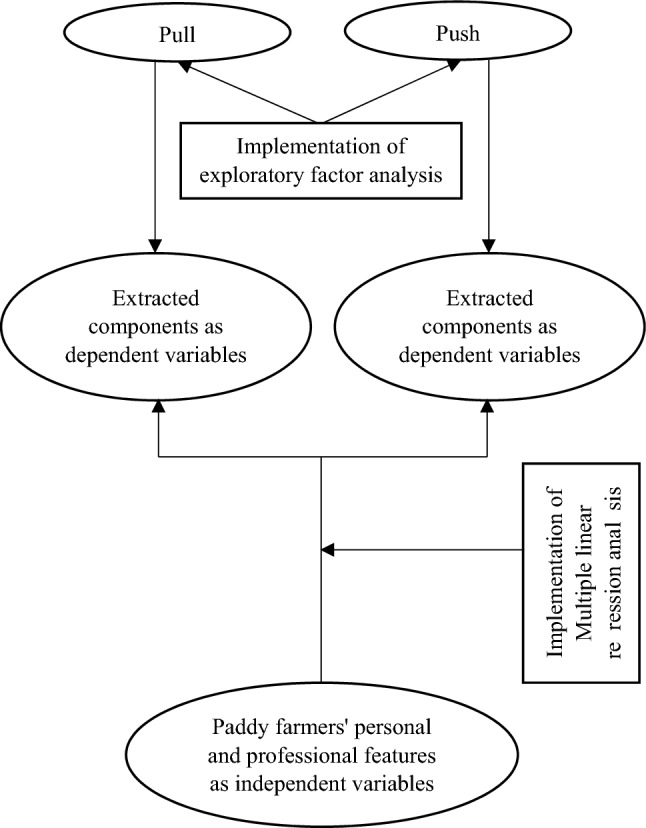
Diagram of the analysis process.

The participants were asked to identify the importance of the 20 possible motives (pull and push) on a five-point scale ranging from ‘‘not important’’ (1) to ‘‘very important’’ (5). Descriptive and inferential statistics were used to analyze the data. In order to extract the underlying components of the pull and push motives, exploratory factor analysis was performed separately. A principal factor analysis with the varimax rotation was performed on the rankings assigned by the paddy farmers in 13 pull motives and 7 push motives related to pluriactivity. Eigenvalues over one and the loadings over 0.50 were the thresholds used in the factor analysis. Cronbach’s alpha reliability analysis was performed to examine the variables’ internal consistency of comprising components. Six pluriactivity motives’ components produced by the factor analysis were considered as dependent variables. Multiple linear regression analysis was then performed to determine the degree of association between PPFs’ personal and professional features (independent variables) with the pluriactive motives’ components (Fig. 3). Finally, the data analysis was implemented using SPSS version 24.

### Statement

All candidates were informed about data protection issues by the enumerators and gave their consent orally at the beginning of each interview. All materials and methods are performed in accordance with the instructions and regulations and this research has been approved by a scientific committee at Tarbiat Modares University (TMU), Iran.

### Informed consent

Informed consent was obtained from all individual participants whose views and opinions are used in this study.

## Results

### Description of the pull and push measurement items

The mean scores and standard deviations of suggested motives used to capture farmers’ motives for starting a new business along with the paddy farming, are shown in Table 2. The results revealed a broad range of economic, social, intrinsic and market-related motives related to pluriactivity decisions within the pull and push typologies. The strongest and weakest motives in the pull typology were independence/self-employment, and the market demand, respectively. Also, among the motives related to push typology, the strongest and weakest ones were unemployment of the family members and reducing debts attributed to paddy farming, respectively.

**Table 2:** Descriptive statistics of suggested motives for starting a new business along with paddy farming.

Suggested motives (n = 182)	Importance mean^a^	Standard deviation
Pull motives		
Independence and self-employment	3.81	1.04
Creating financial sustainability for the future of children (son/daughter)	3.79	1.09
Achieving a better social status	3.68	1.10
Creating jobs at the regional level	3.60	0.99
Creating new social interactions	3.55	0.98
Achieving enough knowledge and experience	3.49	0.93
Continuing the family traditions in business	3.27	1.15
Testing a new business idea	3.21	1.10
Little financial requirements to start the new business	3.13	1.08
Providing new products and services for customers	3.12	1.05
Easy procedure for acquiring the permissions for setting up the business	3.09	1.16
Optimal use of available resources related to paddy farming	3.03	1.09
Market demand	2.98	1.06
Push motives		
Unemployment of family members	3.69	1.08
Continuing the paddy farming and preventing a change in lands’ use	3.67	1.05
Unacceptable economic situation of the paddy farming	3.64	1.11
Lack of income fluctuations’ adjustment throughout the year	3.23	0.99
Strengthening the economic situation of paddy farming (in order to provide agricultural inputs)	3.22	1.13
Alleviation of disasters’ impact	3.03	1.04
Reducing the extent of debts required for the paddy farming (including bank and personal loans)	2.87	1

^a^Five-point scale with 1 = not important to 5 = very important.

### Pull typology

Table 3 shows the three obtained labelled components, the motives loaded in each component and their corresponding loadings, the Cronbach’s alpha reliability coefficients, eigenvalues, and the variance percentage explained by each component in the pull typology. In this typology, a factor solution with three components was retained. The KMO of the final matrix was 0.766. Three items (motives) with lower factor loadings were removed from two steps, starting with the one that had the lowest communality and only significant factor loadings remained. The overall measured reliability was 0.73 and finally, ten motives were loaded in three components. Each of the three components was assigned with a label based on the nature of the loaded motives. These components totally accounted for 58.38% of the variance.

**Table 3:** Rotated factor matrix of the pull motives measured for pluriactivity.

Pull components	Factor loadings	% of explained variance	Initial Eigenvalues
C1. Personal aspirations and pursuit (α = 0.74)^a^		26.03	3.08
Continuing the family tradition in the business	0.66		
Creating new social interactions	0.63		
Achieving a better social status	0.75		
Creating financial sustainability for the future of the children	0.72		
Being independence and self-employment	0.71		
C2. Proper conditions and facilities (α = 0.58)		16.52	1.56
Easy acquiring the permissions for setting up the business	0.80		
Low initial capital requirement	0.79		
Optimal use of available resources related to paddy farming	0.53		
C3. Growth and service markets (α = 0.59)		15.84	1.20
Market demand	0.84		
Providing new products and services for customers	0.62		
% of total explained variance		58.38	
Extraction method: Principal component analysis	KMO = 0.761		
Rotation method: Varimax with Kaiser normalization	Bartlett’s Test of Sphericity = 370.579**		

**99% confidence level.

^a^Cronbach’s alpha reliability coefficients for the components. Overall reliability (α = 0.73).

The first component, “Personal Aspirations and Pursuit (PAP = C1)”, captured 26.03% of the variance related to the set of loaded motives in the pull typology, with an eigenvalue of 3.08 and an alpha reliability coefficient of 0.74. Five motives loaded in this component were related to personal desires. The second component was “Proper Conditions and Facilities (PCF = C2)”. This component mainly comprised of the motives related to required facilities' accessibility for setting up a business along with the paddy farming. This component accounted for 16.52% of the variance, and had an eigenvalue of 1.56 and an alpha reliability coefficient of 0.58. The motives loaded in this component included easy acquiring permissions for setting up the business, low initial capital requirement, and optimal use of available resources related to paddy farming (e. g. machinery equipment, etc.). Two motives loaded in GSM (C3)" were mostly related to “supply and demand in the market”, and “providing new products and services to customers”. This component captured 15.84% of the variance from the set of loaded motives in the pull typology and had an eigenvalue of 1.20. The alpha reliability coefficient calculated for this component was 0.59.

### Push typology

Table 4 illustrates three obtained labelled components, the motives loaded in each component, and their corresponding loadings, the Cronbach’s alpha reliability coefficients, eigenvalues, and variance percentage explained by each component in the push typology. In this typology, a factor solution was derived with three components just similar to the pull typology. The KMO of the final matrix was 0.561; while, the overall measured reliability was 0.57. In sum, seven motives loaded in these three components accounted for 67.97% of the pluriactive motives’ variance. Each of the three components was assigned a label based on the nature of the loaded motives.

**Table 4:** Rotated factor matrix of the push motives measured for pluriactivity.

Pushing components	Factor loadings	% of explained variance	Initial Eigenvalues
C4. Financial status and job creation improvement (α = 0.64)^a^		25.80	2.06
Unemployment of family members	0.89		
Unacceptable economic situation of the paddy farming	0.72		
Continuing the paddy farming and prevention of the land use changes	0.64		
C5. Uncertainty and risk mitigation (α = 0. 68)		22.45	1.51
Adjustment of income fluctuations throughout the year	0.86		
The alleviation of disasters’ impact	0.85		
C6. Paddy farming economic enhancement (α = 0.50)		19.72	1.19
Reducing debts in the paddy farming (including bank and personal loans)	0.85		
Strengthening the economic situation of the paddy farming (in order to better provide agricultural inputs)	0.72		
The total explained variance percentage		67.97	
Extraction method: Principal component analysis	KMO = 0.56		
Rotation method: Varimax with Kaiser normalization	Bartlett’s Test of Sphericity = 210.701**		

**99% confidence level.

^a^Cronbach’s alpha reliability coefficients for components. Overall reliability (α = 0.57).

The component of “improving financial status and job creation” (FSJCI = C4) was mainly in connection with the motives pertaining to “job creation and economic situation improvement”. The motives loaded in this component were “unemployment of family members”, “unacceptable economic situation in the paddy farming” (mainly due to the limited rice cultivation area in each household), and “continuing rice cultivation and preventing the land use changes”. This component captured up to 25.80% of the variance, had an eigenvalue of 2.06 and an alpha reliability coefficient of 0.64. Two motives labelled as URM (C5) were associated with “income fluctuations’ adjustment throughout the year”, and “the alleviation of disasters’ impact” (drought, pests, diseases, etc.). These two motives accounted for 22.45% of the variance from the set of loaded motives of the push typology and had an eigenvalue of 1.51 (Cronbach’s α = 0.68). The last component, which explained 19.72% of the push typology variance, was labeled as “paddy farming economics enhancement” (PFEE = C6). The set of motives within this component included “debts’ reduction” (including bank and personal loans) in the paddy farming, and “strengthening the economic situation of paddy farming” (in order to better supply agricultural inputs). The calculated eigenvalue of this component was 1.19 with an alpha reliability coefficient of 0.50.

### Features correlated to pluriactivity motive components

According to Table 5, multiple linear regressions were performed concerning six pluriactivity motive components. Two statistically significant models were suggested in which PPFs' personal and professional characteristics have affected on the motives, which stimulate the paddy farmers to set up and develop a business along with their paddy farming.

**Table 5:** Multiple linear regressions of PPFs' characteristics of the pluriactivity motive components.

Independent variables	Pluriactivity motives' components (standardized β and significance)					
	Pull components^a^			Push components^b^		
	C1	C2	C3	C4	C5	C6
The age of the pluriactivity initiation as the owner (year)	− 0.28**	0.11	0.03	− 0.24**	− 0.07	0.09
Owner’s paddy farming experience (year)	0.22**	− 0.08	− 0.02	0.13	0.01	− 0.12
Paddy field size (Hectare)	0.21**	0.05	− 0.01	0.21**	− 0.03	− 0.06
Experience at the beginning of pluriactivity as the owner (year)	− 0.09	− 0.02	0.01	− 0.05	− 0.01	− 0.09
Educational level	0.10	− 0.07	0.13	0.13	0.02	− 0.08
*P* value	0.00	0.74	0.63	0.00	0.97	0.32
*R*	0.40	0.12	0.14	0.35	0.07	0.18
*R*^2^	0.16	0.02	0.02	0.12	0.01	0.03
Adjusted *R*^2^	0.14	− 0.01	− 0.01	0.10	− 0.02	0.01

*P ≤ 0.05. **P ≤ 0.01.

^a^C1. Personal aspirations and pursuit; C2. Proper conditions and facilities; C3. Growth and service markets.

^b^C4. Financial status and job creation improvement; C5. Uncertainty and risk mitigation; C6. Paddy farming economic enhancement.

The first statistically significant model showed that the age of pluriactivity initiation as the owner (β =− 0.279, *P* ≤ 0.01), owner's paddy farming experience (β = 0.219, *P* ≤ 0.01), and paddy field size (β = 0.212, *P* ≤ 0.01) are statistically related to the motives loaded in PAP component (C1). These variables explained 15.9% (R^2^ = 0.159, *P* < 0.00) of the variance of PAP component. Second statistically significant model explained 12.3% (R^2^ = 0.123, *P* < 0.00) of the variance of FSJCI component (C4), which included the variables of farmer's age of the pluriactivity initiation as the owner (β =− 0.241, *P* ≤ 0.01), and the paddy field size (β = 0.206, *P* ≤ 0.01). It should be also noted that there was no statistically significant relationship among other motive components with the PPFs’ personal and professional characteristics.

### A comprehensive framework

**Figure 4:**
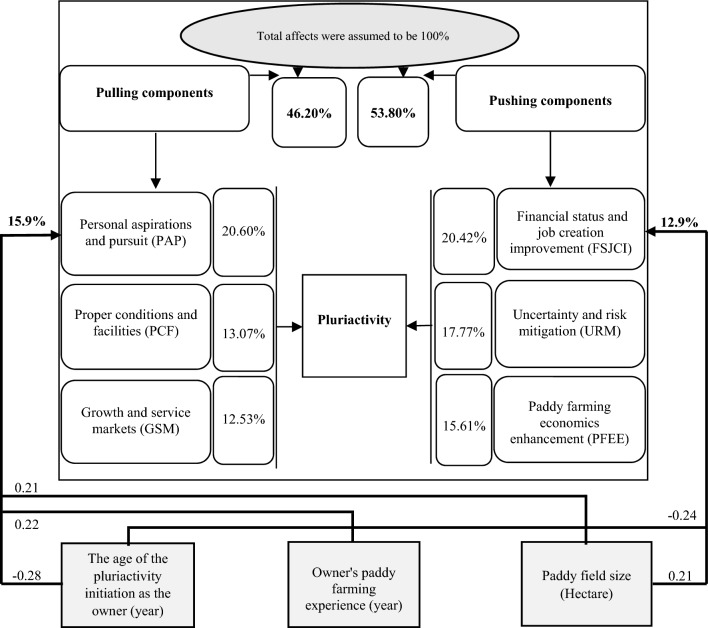
A comprehensive motivational framework to drive paddy farmers towards pluriactivity.

To explain the results more clearly, a general model was presented. To do so, the total effects (pull and push) were assumed equal with 100 and the contribution percentage of each component was calculated on this basis. According to Fig. 4, the pull components contributed 46.20%, including PAP (20.60%), PCF (13.07%) and GSM (12.53%). The push components contributed 53.80%, which were composed of FSJCI (20.42%), URM (17.77%) and PFEE components (15.61%). As mentioned earlier, farmer's age of the pluriactivity initiation as the owner, his/her paddy farming experience, and the paddy field size, explained 15.9% of the variance of PAP component. In addition, farmer’s age of the pluriactivity initiation as the owner, and the paddy field's size explained 12.3% of the variance of FSJCI component.

## Discussion

Based on the results of exploratory factor analysis, each of the pull and push motive typologies were categorized into three components. The most important motives related to PAP were “achieving a better social status”, “creating financial sustainability for the future of the children”, and “being independent”. Achieving better social status has been also considered by Taylor and McClintock^23^ and Blad^29^. Obviously, every human being tends to have an appropriate social status in the society. Some paddy farmers have recognized that starting up their own business is the best way to achieve appropriate social status and more actively participate in social actions. Another motivational item to launch a business is creating financial sustainability for the future of children, which was underlined by Taylor and McClintock^23^ as well. In today's rapidly changing world, we can expect everyone including the paddy farmers to seek financial stability for the future of their families. In this way, they can help their children in the early stages of their independent life to afford a secure source of income for themselves. Being independent and self-employed was another motivation for starting extra business by PPFs. In general, if the paddy farmers need supplementary occupation (for any reason), they tend to setup and manage their own business rather than being dependent to others. Therefore, being dependent and working under the supervision of another person may be unacceptable and unbearable for a great number of the farmers' youth.

As the PCF component, some paddy farmers acknowledged that getting permissions easily is very effective in starting a business. Therefore, this can be a matter of lawmakers' function to facilitate the licensing procedure of the businesses in rural areas without involving entrepreneurs in complex bureaucratic processes. Similarly, some farmers are encouraged to start a business only because of low initial capital requirement.

Market demand is the most important item in GSM component. This motivation has been also addressed in the research of Bowler et al.^61^, Nickerson et al.^62^, Barbieri and Mahoney^26^. Sometimes in a particular geographical area, during a medium and long term, and even short term, there could be a public demand for a specific product that encourages many people to supply that product or service. Considering the seasonality of paddy farming and also noble understanding of the market demand, some paddy farmers are encouraged to set up another business. Oppositely, the necessity of a product or service might be intangible in the community; nevertheless, some intelligent people comprehend this invisible need and offer the required product and/or service, which is generally welcomed by the target community. Similarly, some of the paddy farmers who have a good understanding of the existing intangible needs, launch related businesses. In fact, PPFs offer a new product or service in the region. This motivation has been addressed by Barbieri and Mahoney^26^ as well.

As the push typology, “unemployment of family members”, “unacceptable economic situation of the paddy farming”, and “continuing the paddy farming” and “prevention of land use changes” were classified in the form of FSJCI component. As a result of descriptive statistics, job creation is presumed as the most significant reason and motivation for the business creation and development. This motivation have been considered by Bowler et al.^61^, Sofer^22^, Nickerson et al.^62^, McGehee and Kim^63^, Barbieri and Mahoney^26^, Hansson et al.^27^ in the research projects. As mentioned earlier, the paddy farmers and their families are engaging in that activity over a part of the year (less than 6 months). In other words, they are unemployed in most part of the year. Thus, creating a business can greatly fulfill the unemployed gap of the paddy farmers and their families. Dissatisfactory economic situation of the paddy farming is another effective factor in business development. This motivation has been addressed by Bowler et al.^61^, Sofer^22^, Nickerson et al.^62^, McGehee and Kim^63^, Hansson et al.^27^, Blad^29^ in their studies. Relying solely on the paddy farming (with average area of approximately one hectare) is not economically justifiable and due to that shortcoming some paddy farmers, particularly populous paddy farmers’ families, may face numerous financial problems in handling their living costs. Consequently, the creation of a business is crucial to compensate the income gap among the paddy farmers. Another motive is the paddy farming continuation and prevention of land use changes. This motivation has been pinpointed in the research of Barbieri and Mahoney^26^, Hansson et al.^27^. The rice production and preservation of paddy field as a family legacy inherited from the past generation, are significant for the paddy farmers, both spiritually and emotionally.

Adjustment of the income fluctuations throughout the year, addressed by Nickerson et al.^62^, McGehee and Kim^63^, Barbieri and Mahoney^26^, Hansson et al.^27^, and the alleviation of disasters’ impact, reported by Barbieri and Mahoney^26^ and Hansson et al.^27^, were classified in the component of uncertainty and risk mitigation. The seasonality nature of the paddy farming could strengthen the adjustment goal of income fluctuations throughout the year. Disasters as an unexpected phenomenon are closely connected with the impact of political and climate changes’ dimensions. In terms of the policy making, the government has not been able to appropriately support paddy farmers for various reasons. These supports' shortages could occur in planting stage (lack of support in providing equipment and facilities for planting, etc.), maintenance stage (lack of support in providing necessary inputs, etc.), harvesting stage (lack of support in providing equipment and facilities to harvest the crop) and post-harvest stage (lack of support in providing proper context for selling their products). Climatically, the floods, droughts, pests and diseases can also be noted as uncertainties and risks in agriculture, which have been alternatively happened in the recent years. The paddy farmers sometimes experience floods at the beginning of the farming season (e.g. March and April floods occurred in the year 2019), and sometimes they face with drought (e.g. the droughts occurred from 1996 to 2002), and occasionally have to deal with pest and disease attack at the maintenance and beginning of the paddy harvesting stages. These natural disasters lead to drastic reductions in both quantity and quality of the rice products. So, in order to minimize the impact of negative aspects of mentioned uncertainties, it is obviously logical for the paddy farmers to have another source of income along with the paddy farming.

Reducing the debts in the form of PFEE, which has been addressed by Bowler et al.^61^, Sofer^22^, Nickerson et al.^62^, McGehee and Kim^63^, Barbieri and Mahoney^26^, Hansson et al.^27^ is of great importance too. Preparing and supplying the facilities and inputs at different stages of planting, and also maintaining and harvesting the paddy crops requires sufficient financial resources. Nonetheless, many paddy farmers are not able to provide the required funding for different reasons; hence, they have to get loans from several banks and financial institutions. Sometime, this situation may continue for several years and it imposes a lot of debts to the paddy farmers. Therefore, some paddy farmers would start an additional business to be able to afford the past undertaken debts. Also, a number of the paddy farmers are forced to startup a new business in order to strengthen their economic status and could supply required rice inputs (such as fertilizers, pesticides, farm machinery, labor costs). This motivation has been mentioned in the research accomplished by Hansson et al.^27^.

The regression analysis showed that two models were substantial out of six components. The first model uncovered the fact that those paddy farmers who have engaged in pluriactivity at a younger age and have more paddy farming experience and larger paddy field size, are more likely to pursue personal aspirations. On the other hand, through engaging in diversification activities, they seek to be able to better provide financial sustainability for their children's future. The fourth model illustrated that the farmers who are involved in diversification activities at a younger age and possess greater paddy field size, are more likely to have a stronger incentive for strengthening the financial status and job creation. Therefore, by diversified activities, they seek to generate higher income and use it in paddy farming. With this motivation, PPFs certainly seek to create financial sustainability for their businesses. In fact, through this procedure the farmers can create a long-lasting financial cycle for years. As such, these capacities provide a trustable basis for sustainable employment of family members, continuation of the paddy farming and prevention of the land use changes. The noteworthy point is that in this study, the effect of educational level on the six components of motivation is not significant. This result is in line with Niemela's^30^ research and contrary to Martinez^28^, Delmar^33^ and Morris’s^55^ research. Considering that most of the launched businesses are indigenous and not technologically advanced and sophesticated, it can be concluded that the establishment of rural businesses in the study area does not depend to a large extent on the level of education.

In general, promoting rural resilience through pluriactivity plays a major role in sustainable rural development. The development of pluriactivity improves the livelihood and living standards of the people in rural areas, which in the long run makes urban and rural relations more coherent and reduces the risk of decline of rural communities. Therefore, by knowing the capacities of rural communities and motivations of rural people, it is possible to take advantage of the economic potentials and entrepreneurial opportunities in rural areas for the materialization of a dynamic and diverse economy.

## Conclusions and policy implications

According to Panarchy theory^68^, as one of the components of resilience, by combining the side and main activity, which is called pluriactivity, a balanced economic, social and environmental cycle is created for the rural communities, which in turn causes rural resilience. For example, in the period that rice farming does not perform well due to bad weather conditions or pest infestation, the rice farmer's family suffered considerably losses. In other words, while, the economic shock could inflict on the farmer's household, the existence of side activities and earning money from them can moderate the economic shock to a large extent and help farmers and ultimately the rural community to appropriately survive. Thus, pluriactivity as a livelihood strategy can play an effective role in rural resilience; meanwhile, motivation is considered as the central force of the paddy farmers' pluriactivity. The motivation criteria were classified into the pull and push typologies. It seems that the pull typology motives were more focused on personal/professional growth aspect; though, the push typology motives were more concentrated on economic side. Consequently, it is imperative to precisely consider both pull and push typologies in directing rural societies in general and paddy farmers in particular toward extension and the development of entrepreneurial businesses in rural areas. In addition, attention should be paid to the motives with spiritual aspect of the paddy farmers in the study region for materializing rural business development. According to the need and interest principle^69^, a new business would be more likely sustainable, when a person starts it based on his/her own personal interest and without any external pressure. It is also worth noting that in rural areas, including the study region, the paddy farmers occasionally establish a new venture along with their paddy farming activity due to low prices of agricultural crops (rice) on the one hand and high costs of agricultural inputs with inevitable economic and financial pressure (push typology) on the other hand. When farmers start a new business because of economic and financial pressures, they more possibly face numerous obstacles, including bank debts, market problems, and so on. Accordingly, providing an appropriate and comprehensive supportive framework (policy, economy, market, etc.) will be very effective in sustaining established businesses and resolving paddy farmers' problems. Therefore, the sustainability of a business, in particular for the paddy farmers, would create a maintainable financial and business cycle. In another part of the study, the results revealed that the age, farm size and experience are effective in paddy farmers’ motivation to diversify their businesses. It can be concluded that in both pull (specifically the component of PAP) and push (particularly the component of strengthening financial status and job creation) motives, age and paddy farming experience are of great importance. Therefore, in order to persuade the farmers' community in general and the paddy farmers in particular, taking these variables into account can be quite operational for the creation and development of additional rural businesses.

In the field of pluriactivity, unlike other studies, this research underlined that the typologies of pull and push motives are different in nature and each of them have different effects on starting an extra business. On the other hand, application of our findings by policymakers will be a fundamental step in creating and developing sustainable businesses in the region. However, under different conditions (place, time, and subjects) one can consider a number of other motives involved in diversification and pluriactivity. Ultimately, one of the main limitations of this study was dealing with anonymous statistical population and using non-probability sampling method, which makes generalization of the findings difficult. Hence, it is necessary to consider regional characteristics and demographics of the population when adopting this formula. Nowadays, rural businesses in the study area have a great variety^70^. The newest rural businesses in the study area, are extending due to mechanization development in paddy cultivation, agricultural services including Transfer Planting Bank (TPB) and Agri-drones, which are considered as advanced and sophisticated agricultural methods. Rice farmers are more or less willing to use these services, but very few people provide services in these two areas. The services of the TPB include seedling production services (for indirect cultivation of the rice in irrigated lowland conditions), tractor rental, and seeding machine rental, etc. Also, the services of agricultural drones consist of spraying fertilizers and pesticides, fungicides and herbicides, field monitoring, and field mapping, etc. On the other hand, most of the children (youths) of the paddy farming families have higher education, but do not have suitable jobs. Consequently, taking into consideration the findinggs of this research characteristics of the region, and demographics in the study site, the stakeholders should take steps towards the development of rural businesses.

## Data Availability

The datasets generated and/or analyzed during the current study are not publicly available (because all of the data was gathered by the research team) but are available from the corresponding author on reasonable request.
